# Supplementary material for: AffyMAPSDetector: a software tool to characterize Affymetrix GeneChip™ expression arrays with respect to SNPs
Source: BMC Bioinformatics. 2007 Jul 30;8:276. doi: 10.1186/1471-2105-8-276 (PMC1959249; doi:10.1186/1471-2105-8-276)
Supplement: Additional file 13 — AffyMAPSDetector v1 source code. AffyMAPSDetector source code. [file 1471-2105-8-276-S13.zip › AffyMAPSDetector_v1_src/doc/jwf/WizardUtils.html]

WizardUtils


|  |  |  |  |
| --- | --- | --- | --- |
| **Overview** \| **Package** \| **Class** \| **Use** \| **Tree** \| **Deprecated** \| **Index** \| **Help** | | |  |
| **PREV CLASS**   NEXT CLASS | **FRAMES**    **NO FRAMES**     **All Classes** |  |  |
| SUMMARY: NESTED \| FIELD \| CONSTR \| METHOD | DETAIL: FIELD \| CONSTR \| METHOD |  |  |


---


## jwf Class WizardUtils

```
java.lang.Object
  jwf.WizardUtils
```

---

public class **WizardUtils** extends java.lang.Object

Description: This is a general purpose utility class that is a
convenient place for wizard-wide utitilites.

Copyright: Sunita Kumari -- Copyright (c) 2004

Company: George Mason University

**Version:**
:   1.1

**Author:**
:   Sunita Kumari

---

|  |  |
| --- | --- |
| **Field Summary** | |
| `static int` | `MAX_LABEL_WIDTH` |


**Constructor Summary**

`WizardUtils()`


|  |  |
| --- | --- |
| **Method Summary** | |
| `static java.lang.String` | `getFixedWidth(java.lang.String label)` |
| `static java.lang.String` | `getFixedWidth(java.lang.String label, int maxWidth)` |
| `static void` | `setLabelFont(javax.swing.JLabel label)` |
| `static java.io.File` | `showFileChooser(java.awt.Component parent)`             This method should be used to show if file system needs to be browsed. |

**Methods inherited from class java.lang.Object**

`clone, equals, finalize, getClass, hashCode, notify, notifyAll, toString, wait, wait, wait`

**Field Detail**

### MAX\_LABEL\_WIDTH

```
public static final int MAX_LABEL_WIDTH
```

**See Also:**: Constant Field Values


**Constructor Detail**

### WizardUtils

```
public WizardUtils()
```


**Method Detail**

### getFixedWidth

```
public static java.lang.String getFixedWidth(java.lang.String label)
```

---


### getFixedWidth

```
public static java.lang.String getFixedWidth(java.lang.String label,
                                             int maxWidth)
```

---


### setLabelFont

```
public static void setLabelFont(javax.swing.JLabel label)
```

---


### showFileChooser

```
public static java.io.File showFileChooser(java.awt.Component parent)
```

:   This method should be used to show if file system needs to be browsed.

    :   **Parameters:**: `parent` - type java.awt.Component -- parent component that would show file chooser dialog. **Returns:**: type java.io.File -- selected file, null if no file is selected


---


|  |  |  |  |
| --- | --- | --- | --- |
| **Overview** \| **Package** \| **Class** \| **Use** \| **Tree** \| **Deprecated** \| **Index** \| **Help** | | |  |
| **PREV CLASS**   NEXT CLASS | **FRAMES**    **NO FRAMES**     **All Classes** |  |  |
| SUMMARY: NESTED \| FIELD \| CONSTR \| METHOD | DETAIL: FIELD \| CONSTR \| METHOD |  |  |


---
